# The mouth‐opening muscular performance in adults with and without temporomandibular disorders: A systematic review

**DOI:** 10.1111/joor.13303

**Published:** 2022-01-31

**Authors:** Tzvika Greenbaum, Laurent Pitance, Ron Kedem, Alona Emodi‐Perlman

**Affiliations:** ^1^ Department of Physical Therapy Faculty of Health Sciences Recanati School for Community Health Professions Ben‐Gurion University of the Negev Beer Sheva Israel; ^2^ Institute of Experimental and Clinical Research Health Sciences division Neuro‐Musculo‐Skeletal‐Lab (NMSK) Université Catholique de Louvain Brussels Belgium; ^3^ Academic Branch Medical Corps IDF Tel Aviv Israel; ^4^ The School of Dental Medicine Sackler Faculty of Medicine Tel Aviv University Tel Aviv Israel

**Keywords:** jaw‐opening force, mouth‐opening force, muscle strength, suprahyoid muscle, temporomandibular disorders, temporomandibular joint

## Abstract

**Background:**

The mouth‐opening muscular performance in patients with temporomandibular disorders (TMDs) is unclear. Understanding the impairments of this muscle group within specific TMDs is important to develop proper management strategies.

**Objective:**

To characterise the mouth‐opening muscular performance in adults with and without TMDs.

**Methods:**

PubMed, EMBASE, CINAHL, Scopus, Web of Science and Cochrane databases were searched from inception to 12 November 2020. Bibliographies were searched for additional articles, including grey literature. Case‐control, cross‐sectional and interventional studies reporting mouth‐opening muscular strength and/or endurance were included. Risk of bias was assessed by the SIGN checklist for case‐control studies and by the NIH quality assessment tool for cross‐sectional studies. Results were pooled with a random‐effects model. Confidence in cumulative evidence was determined by means of the GRADE guidelines.

**Results:**

Fourteen studies were included; most were rated as having a moderate risk of bias. Only three studies assessed patients with TMDs and the other 11 assessed healthy adults. Significant sex differences in muscular performance were found for healthy adults in the review (strength deficit for females versus males). There was a significant reduction in maximal mouth opening performance (strength and endurance) in the three studies that assessed patients with temporomandibular disorders.

**Conclusion:**

Sex plays a significant role in maximal mouth opening strength. There is a lack of reliable data on the normal mouth‐opening strength and endurance of healthy adults as well as for patients with TMDs.

**Implications:**

Lack of reliable TMDs patient data and comparable healthy adult data highlight future direction for research.

## Graphical abstract

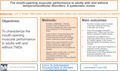

## 1 INTRODUCTION

The masticatory muscles are divided into two main categories according to their functions of mouth openers or mouth closers.^1^ The mouth closers are the masseter, temporalis and medial pterygoid muscles which work against gravity and are more dominant and stronger than the mouth openers.^1^ They are, therefore, considered as one of the most common sites of pain in the masticatory system.^1^ The mouth closers are also closely involved in both awake and sleep bruxism (masticatory muscle activity during sleep or wakefulness).^2^ The main opener muscle of the mouth is the lateral pterygoid muscle, which also contributes to protrusion and lateral deviation of the mandible, both of which are movements required for normal mastication.^3^ The other mouth opening synergists are the supra‐ and infra‐hyoid muscles, which are also involved in different oromotor functions, such as tongue stability, swallowing and speech.^1^ There are four suprahyoid muscles on each side of the mouth, the stylohyoid, digastric, mylohyoid, and geniohyoid, and two infrahyoid muscles on each side of the anterior neck, the sternohyoid and omohyoid.

The muscular performance of the mouth closers has been intensively researched in both healthy controls and patients with temporomandibular disorders (TMDs).^4^, ^5^, ^6^, ^7^ In contrast, comparable knowledge on mouth openers is very limited. A recent systematic review and meta‐analysis which assessed the muscular function of patients with TMDs observed that no study that measured the function of the mouth openers had been included compared to 22 studies that evaluated the function of the mouth closers.^8^ The most widely researched population among the few available studies that did assess the muscular performance of the mouth openers comprised healthy elderly individuals from Japan.^9^, ^10^, ^11^ One of the main reasons given for under‐researching the mouth openers is that activation of the mouth opening muscles is not required for the initial phase of functional mouth opening but rather relaxation of the mouth closers.^1^ This argument is mainly valid for the initial phase of mouth opening but not for common masticatory muscle functions, such as yawning, or even gum chewing that requires muscular activation of the mouth openers.^12^ Furthermore, given that patients with TMDs are very likely to present with over‐activity of the mouth closers,^2^ it could be hypothesised that their mouth openers are also required to be active during the initial phase of mouth opening in order to overcome the actions of the closers. It is also very likely that, similar to other regions of the human body, the relationship between the muscular agonist‐antagonist is a relevant factor in rehabilitation of the associated musculoskeletal disorders.^13^, ^14^

The aim of this review was to systematically evaluate the currently existing evidence on the muscular performance of the mouth openers in patients with TMDs. The research questions were as follows:

1. What is the normal range of human mouth‐opening muscular performance (strength and endurance)?
2. Are there standardised, valid and reliable tests to measure mouth‐opening muscular performance (strength and endurance)?
3. Is mouth‐opening muscular performance (strength and endurance) impaired in patients with TMDs compared to healthy controls?

## 2 METHODS

A review protocol was developed according to the Preferred Reporting Items for Systematic Reviews and Meta‐analyses (PRISMA)^15^ and registered with Prospero prior to initiating this systematic review (Registration date: Dec 15, 2020, CRD42020220878).^16^

### 2.1 Identification and selection of studies

**FIGURE 1:**
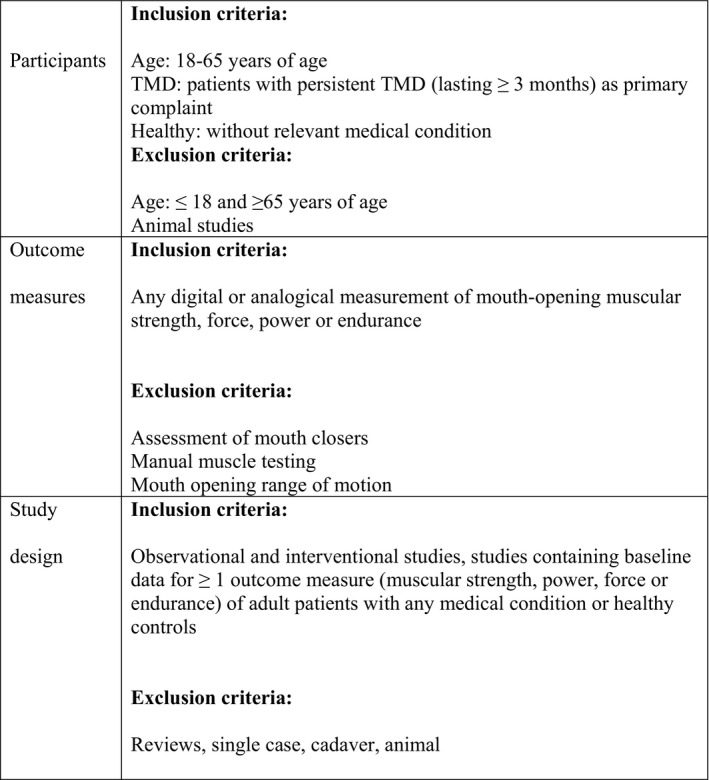
Eligibility criteria for systematic review

PubMed (MEDLINE), EMBASE, CINAHL, Web of Science, Scopus and Cochrane Central databases were searched by one reviewer (TG) to identify potentially relevant articles. The search strategy and number of identified studies for each database are listed in Table 1. Reference lists from the included studies were also scanned to identify additional relevant studies. No restriction was placed on publication date. Studies identified by the search were transferred to Endnote X9 (Clarivate Analytics) and duplicates were removed. The remaining studies were then uploaded into Covidence systematic review software (Veritas Health Innovation) where two independent reviewers (TG and AEP) screened the titles and abstracts to identify potentially eligible articles. The full texts of the remaining studies were retrieved for further assessment and were included/excluded according to the eligibility criteria (Figure 1). Reasons for exclusions during full‐text screening were recorded for future reference. All stages of the screening and assessment were performed independently by the two reviewers, and meetings were held periodically to compare and discuss decisions. In the case of disagreement, a third review member was consulted (LP).

**TABLE 1:** Search strategy (all databases); date of all searches: Nov. 11, 2020

Database	Search strategy	Number of identified record
Embase	(‘mouth opening’ OR ‘jaw opening’ OR suprahyoid* OR ‘supra hyoid*’) AND (strength OR force$ OR power OR endurance)	694
MEDLINE	(("mouth opening" or "jaw opening" or suprahyoid* or ‘supra hyoid*’) and (strength or force? or power or endurance)).mp.	500
CINHAL	("mouth opening" OR "jaw opening" OR suprahyoid* OR "supra hyoid*") AND (strength OR force# OR power OR endurance)	127
Web of Science	Search Strategy: ("mouth opening" OR "jaw opening" OR suprahyoid* OR "supra hyoid*") AND (strength OR force? OR power OR endurance)	653
Scopus	Search Strategy: TITLE‐ABS‐KEY ("mouth opening" OR "jaw opening" OR suprahyoid* OR "supra hyoid*") AND TITLE‐ABS‐KEY (strength OR force OR power OR endurance)	431
Cochrane CENTRAL	Search Strategy: ("mouth opening" OR "jaw opening" OR suprahyoid* OR ‘supra hyoid*’) AND (strength OR force? OR power OR endurance)	101
Total	2506	
Total after removing duplicates	1051	

### 2.2 Outcome measures

The main outcome measure of this study was muscular performance during mouth opening which included maximal muscle strength and muscle endurance. The secondary outcome measure was muscular performance during mandibular protrusion (a component of full mouth opening) which included maximal muscle strength and endurance.

### 2.3 Data extraction

Following inclusion into this analysis, data were extracted from each study by means of a standardised form, which had been developed and agreed upon by the review team (see Appendix 1). The information extracted from each study included: (a) study characteristics (design, sample size, recruitment methods, inclusion/exclusion criteria), (b) participant characteristics (age, sex, TMDs and other related oro‐facial dysfunctions), (c) diagnostic methods (tools/criteria used to diagnose and classify TMDs and other related oro‐facial dysfunctions) and (d) study outcome measures (measurement tools and testing procedures). Missing or unclear data were annotated as “not specified” or “unsure”, respectively, and the authors of those publications were contacted for clarification. Data collection was performed independently by two reviewers (TG and AEP). Any disagreements were resolved through consultation with a third review member (LP) and the outcome was documented. All forms were stored for future reference. Results for each relevant outcome measure were extracted by one reviewer (TG) and recorded directly into a protected file.

### 2.4 Risk of bias assessment

The risk of bias for each eligible study was evaluated independently by two reviewers (TG and AEP) using two different quality assessment tools. The SIGN checklist^17^ was used for case‐control studies and for interventional studies which included cases and controls (Appendix 2). The NIH quality assessment tool^18^ was used for cross‐sectional studies and for interventional studies which included only one homogeneous group (Appendix 3). The main domains of both quality assessment tools explored (a) sample selection and characteristics, (b) assessor blinding, (c) validity, reliability, and standardisation of outcome measures, (d) confounders and (e) statistical methods. Prior to their implementation, the SIGN and NIH checklist items were discussed by two reviewers (TG and AE) and underwent a pilot assessment to ensure consistency in marking. Each reviewer completed the SIGN/NIH checklist for the included studies and determined an overall risk of bias rating of low (score of 9–12 methodological points), moderate (score of 5–8 methodological points), or high (score of 0–4 methodological points). Intra‐rater agreement was calculated with Cohen's Kappa. Any disagreement was resolved through discussion with a third review member (LP). The authors of the publications were contacted for clarification in the case of unclear or missing information.

### 2.5 Data analysis

The outcome measure data were compared between studies to establish patterns within and/or between the patient populations and control groups. A meta‐analysis was planned to be performed using primary outcome measures where there were ≥5 studies with (a) low to moderate risk of bias and (b) similar assessment and measurement techniques. Results for eligible studies were pooled using Review Manager via a random effects model. Mean differences and standard mean differences were used to determine differences between subgroups, with 95% confidence intervals (CIs) and heterogeneity calculated by means of Cochran's Q test.^15^ Studies with high risk of bias, heterogenous assessment procedures or incomplete statistical reporting (e.g. absence of standard deviation [SD] values) were not included in this meta‐analysis.

#### 2.5.1 Confidence in cumulative evidence

The confidence in cumulative evidence was assessed for each outcome according to GRADE guidelines.^19^, ^20^, ^21^, ^22^, ^23^, ^24^, ^25^, ^26^ Each outcome was given an overall confidence level of “high”, “moderate”, “low” or “very low”, taking into consideration factors, such as risk of bias, consistency of results, effect size and sample size.

## 3 RESULTS

### 3.1 Study selection

**FIGURE 2:**
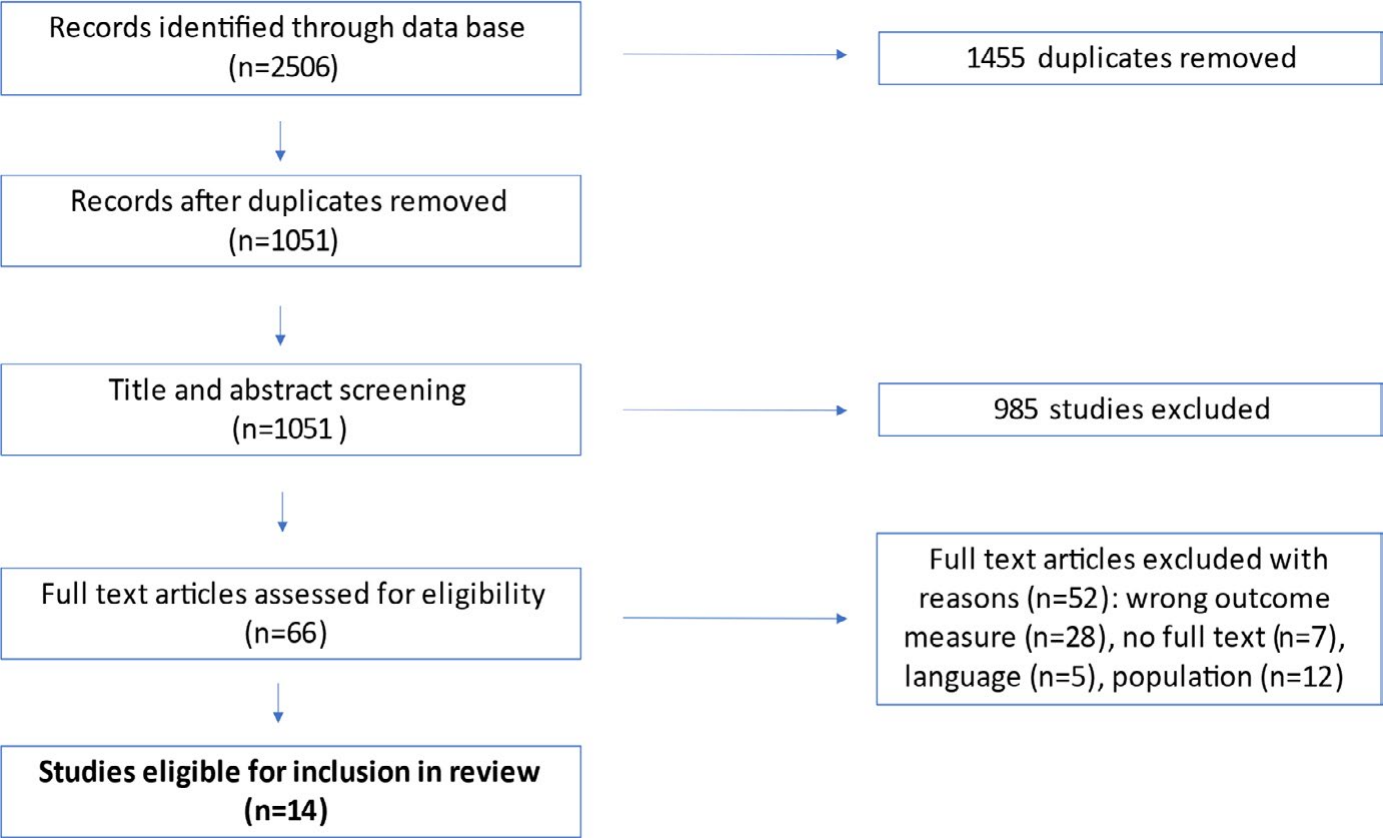
PRISMA flowchart of included and excluded studies

The progression of studies through the review process is demonstrated in Figure 2.

The database search identified 2506 studies, of which 1455 were duplicates. Following screening of titles, abstracts and full texts, 14 studies met the eligibility criteria and were included in this review. The list of full text excluded and the reason for exclusion is shown in Table 2.

**TABLE 2:** list of full text excluded and the reason for exclusion

Study	Reason for exclusion
Nakamura, 2019^38^	Language (Japanese)
Slater, 2009^39^	Population (Cadaver)
Peck, 2000^36^	Outcome measure
Mimura, 1989^37^	Language (Japanese)
Lida, 2014^40^	Abstract only
Rodriguez, 2015^41^	Outcome measure
Pal, 2011^42^	Outcome measure
Chen, 2000^43^	Outcome measure
Ikebe, 2008^44^	Outcome measure
Namiki, 2020^45^	Abstract only
Hara, 2019^45^	Abstract only
Bolt, 1986^46^	Abstract only
Manda, 2016^47^	Outcome measure
Bakker, 1995^48^	Outcome measure
Nagashima, 1997^49^	Outcome measure
Hansdottir, 2004^50^	Outcome measure
Stefanie, 2010^51^	Abstract only
Johansson, 2014^52^	Outcome measure
Takanobu, 2001^53^	Outcome measure
Madani 2020^54^	Outcome measure
Nitzan, 1997^55^	Outcome measure
Abbink, 1998^56^	Outcome measure
De Felicio, 2007^57^	Language (Portuguese)
Clark, 1991^58^	Outcome measure
Koc 2012^59^	Outcome measure
Lin, 2010^60^	Outcome measure
Kilinc, 2015^61^	Outcome measure
Williams, 1988^62^	Outcome measure
Suenaga, 2000^63^	Outcome measure
Kameda, 2020^64^	Outcome measure
Tuijt, 2010^65^	Population (not described)
Wakasugi, 2017^40^	Population (Age)
Van, 1990^66^	Population (Age)
Ishida, 2015^67^	Outcome measure
Ma, 2013^68^	Outcome measure
Uchida, 1999^69^	Outcome measure
Osborn, 1995^70^	Outcome measure
Gelb, 1984^71^	Abstract only
Beom, 2015^72^	Population
Yoshida, 2006^73^	Language (Japanese)
Hara, 2018^74^	Language (Japanese)
Lequeux, 2005^75^	Outcome measure
Oh, 2020^76^	Outcome measure
Peck, 2002^77^	Outcome measure
Chandran, 2012^78^	Abstract only
Machida, 2017^11^	Population (Age)
Hara, 2018^9^	Population (Age)
Yuka, 2020^30^	Population (Age)
Yoshida, 2019^79^	Population (Age)
Yoshimi, 2018^9^	Population (Age)
Kajisa, 2018^10^	Population (Age)

### 3.2 Study characteristics

The characteristics of each eligible study are shown in Table 3. Twelve of the fourteen studies were observational (8 cross‐sectional, 3 case‐control and 1 reliability) and two were interventional (one randomised control trial and two clinical trials). The most frequently used outcome measure was maximal mouth opening strength (12 studies), and only two studies measured muscular endurance.^27^, ^28^ Three studies used the same measurement device and similar testing procedure (jaw‐opening sthenometer by Livert),^9^, ^29^, ^30^ two other studies used similar devices^31^, ^32^ and the remaining nine studies used a specific ad‐hoc unique measurement device with different testing procedures.

**TABLE 3:** Characteristics of included studies

Study	Design	Participants	Measurement instrument	Outcome measure
Brunton, 2018^80^	Cross‐sectional	Healthy (*n* = 149; 98 females; 51 males)	Adjustable rigid extra‐oral device (ad hoc)	Maximal mouth opening force/strength
Curtis, 2019^81^	Cross‐sectional	Healthy (*n* = 216; 129 females; 87 males)	Hand‐held dynamometer	Maximal mouth opening force/strength
Häggman‐Henrikson, 2018^27^	Clinical trial	TMD (*n* = 77; 67 females; 10 males)	Adjustable rigid extra‐oral device (ad hoc)	Mouth opening endurance
Hara, 2018^82^	Cross‐ sectional	Healthy (*n* = 980; 601 females; 379 males)	Jaw‐opening sthenometer (Livert)	Maximal mouth opening force/strength
Lida, 2013^29^	Case control	Healthy (age <70 year; *n* = 76; 38 females); Healthy elderly (age >70 year; *n* = 74; 38 females)	Jaw‐opening sthenometer (Livert)	Maximal mouth opening force/strength
Koyama, 2005^83^	Reliability study	Healthy (*n* = 12; 6 females)	Adjustable rigid extra‐oral device	Maximal mouth opening force/strength
Ogawa, 2020^30^	RCT	Healthy men (*n* = 24 all males)	Jaw‐opening sthenometer (Livert)	Maximal mouth opening force/strength
Ratnayake, 2020^84^	Case control	TMD (*n* = 58; 46 females); Healthy (*n* = 56; 32 females)	Adjustable rigid extra‐oral device	Maximal mouth opening force/strength
Sharkey, 1984^85^	Cross‐ sectional	Healthy (*n* = 55; 20 females)	Adjustable rigid extra‐oral device	Maximal mouth opening force/strength
Takuro, 2018^33^	Cross‐ sectional	Healthy (*n* = 103; 57 females)	Not described	Maximal mouth opening force/strength
Wänman, 2012^28^	Case control	TMD (*n* = 81; 68 females); Healthy (*n* = 75; 54 females)	Adjustable rigid extra‐oral device (ad hoc)	Mouth opening endurance
Watanabe, 1991^31^	Cross‐ sectional	Healthy (*n* = 17 all males)	Adjustable rigid extra‐oral device (ad hoc)	Maximal mouth opening force/strength
Watanabe, 2001^32^	Cross‐ sectional	Healthy (*n* = 26 all males)	Adjustable rigid extra‐oral device (ad hoc)	Maximal mouth opening force/strength
Xu, 2020^86^	Cross‐ sectional	Healthy (*n* = 87; 42 females)	Adjustable rigid extra‐oral device (ad hoc)	Maximal mouth opening force/strength

### 3.3 Participants

A total of 1867 adults were included across the 14 studies (mean age = 39.8 ± 12.0 years). All studies included data on sex which could be pooled, and they yielded 1122 females (60%) and 755 males (40%). The combined study participants were divided into two main subgroups according to their health condition: 1651 healthy controls (mean age = 39.8 ± 12; 57% females) and 216 patients with TMDs (mean age = 37.6 ± 11.6; 83% females).

### 3.4 Outcomes

The studies which evaluated each of the two subgroups are shown in Table 4. Eleven of the fourteen included studies evaluated the mouth opening performance of healthy controls and three of patients with TMDs (two compared to controls and one with TMDs only).

**TABLE 4:** Main findings of included studies

Population	Study	Main findings	Risk for bias
Healthy	Brunton, 2018	Men had greater maximum opening force median values than women; Maximal mouth opening strength: Men 8 ± 6.6 kg; Women 4.2 ± 3.1	Moderate
	Curtis, 2019	Age and sex significantly influenced the mouth opening maximal force; Maximal mouth opening strength: Male 24.9 ± 4.5; Female 14.7 ± 3.2	Moderate
	Hara, 2018	Sex significantly influenced the maximal mouth opening strength (Male >Female); Maximal mouth opening strength: Male 7.2 ± 2.3; Female 4.3 ± 1.7	Moderate
	Koyama, 2005	There was a significant gender difference in the average maximum mouth opening force. There was an extremely high correlation between first and second measurements (*r* = 0.969). Maximal mouth opening strength: Men 24.3 ± 1.3; Women 16.4 ± 1.2	Moderate
	Lida, 2013	Sex significantly influenced the maximal mouth opening strength (Male > Female); Maximal mouth opening strength: Male 9.7 ± 2.8; Female 5.9 ± 1.6 kg Male 9.7 ± 2.8; Female 5.9 ± 1.6 kg	Low
	Ogawa, 2020	Maximal mouth opening strength: Group 1 (pre‐intervention) = 8.7(1.9); Group 2 (pre‐internevtion) = 8.6(1.5)	Moderate
	Ratnayake, 2020	Maximal mouth opening strength: TMD free 4.8 ± 0.15;	Moderate
	Sharkey,1984	Male were significantly stronger than female; Maximun maximal mouth‐opening force accured in the mid‐range Maximal mouth opening strength:Men 13.8 ± 6.1; Women 9.1 ± 2.0	Moderate
	Takuro, 2018	Men were significantly stronger than women; Maximal mouth opening strength: Men 9.2 ± 2.8; Women 6 ± 2.3	High
	Watanabe,1991	The theoretical maximal mouth opening strength was 32.55 ± 4.98	High
	Watanabe, 2001	The theoretical maximal mouth opening strength was 36.62 ± 9.42	High
	Wänman, 2012	Mean time to stop the jaw opening‐closing endurance task: Controls 278 ± 59 (seconds)	High
	Xu, 2020	The median of maximal mouth opening strength was higher in males (5.5) than females (3.4) (*p* < 0.05), but the maximal mouth opening strength were not associated with age, height and weight. Maximal mouth opening strength: Male 5.5; Female 3.4; No SD values provided	Moderate
TMD's	Häggman‐Henrikson, 2018	The "general pain" TMD (according to DC/TMD) group had lower endurance than the "local pain" TMD group (DC/TMD) in both jaw opening and protrusions. No accurate numbers are described but rather only box plots	Moderate
	Ratnayake, 2020	With all five measurements used, this ICC was 0.996 (95% CI: 0.994 to 0.997), indicating extremely high reliability; TMD‐free participants had greater jaw‐opening forces than TMD patients (diagnosed according to DC‐TMD) both without and with adjustments for age, sex, height, and weight; No significant difference between TMD subgroups. Maximal mouth opening strength: TMD patients 1.8 ± 0.16; TMD free 4.8 ± 0.15	Moderate
	Wänman, 2012	Significant lower endurance was found for TMD's (diagnosed according to DC‐TMD) compared to healthy controls. Mean time to stop the jaw opening‐closing endurance task: TMD's 118 ± 96 (seconds); Controls 278 ± 59	High

### 3.5 Risk of bias

Assessment of the risk of bias of each study included in this systematic review is summarised in Table 5a for cross‐sectional or similar study designs and in Table 5b for case‐control or similar study designs. Only one study^29^ was found to have a low risk of bias and nine studies were scored as having a moderate risk of bias. This rating correlated with most of the SIGN^17^/NIH^18^ Quality Assessment Tool checklist criteria having been met, with some flaws in the study resulting in an associated risk of bias. Several studies lacked blinding of the assessors, justification of the sample size and/or reliability of the outcome measure. The main confounders included age, body mass index and sex and of the participants. Four studies^28^, ^31^, ^32^, ^33^ were rated as being at high risk of bias, meaning that either most of the SIGN/NIH Quality Assessment checklist criteria were not met and/or there were significant flaws related to key aspects of the study design. This rating correlated with most of the SIGN/ NIH Quality Assessment Tool checklist criteria not having been met, with significant flaws in the study methodology, resulting in an associated risk of bias. The reliability of risk of bias rating between reviewers was excellent (*κ* = 0.91).

**TABLE 5:** (a) Risk of bias assessment of cross‐sectional studies according to the NIH quality assessment tool. (b) Risk of bias assessment of case‐control studies according to the SIGN quality assessment tool

(a)														
Study	Design	Focused question	Comparable populations	Same exclusion criteria	Comparison participants/ non‐participants	Cases are clearly defined and differentiated from controls	It is clearly established that controls are non‐cases	Blinding of assessors	Exposure status is measured in a standard, valid and reliable way	Confounders are identified and considered	Confidence intervals are provided	Clear association between exposure and outcome	Applicability of study	Risk of Bias
Hֳaggman‐Henrikson, 2018	Clinical trial	Yes	Yes	Yes	No	Yes	No	No	Yes	Yes	Yes	No	No	Moderate (7/12)
Lida, 2013	Case control	Yes	Yes	Yes	No	Yes	Yes	No	Yes	Yes	Yes	Yes	Yes	Low (10/12)
Ratnayake, 2020	Case control	Yes	Yes	No	No	Yes	Yes	No	Yes	Yes	Yes	Yes	Yes	Moderate (9/12)
Wֳanman, 2012	Case control	Yes	Yes	Yes	No	Yes	Yes	No	Yes	No	No	No	No	High (6/12)

(b)														
Study	Design	Research question or objective clearly stated?	Population specified and defined	Participation rate of eligibility (>50%)	Clear inclusion/ exclusion criteria	Sample size justification	Exposure(s) of interest measured prior to the outcome(s) being measured	Timeframe sufficient for association between exposure and outcome	Examination of different levels of the exposure as related to the outcome	Exposure measures definition, validity and reliability	Were the outcome measures definition, validity and reliability	Outcome assessors blinded to the exposure	Key potential confounding variables measured and adjusted statistically	Risk of Bias
Curtis, 2019	Cross sectional	Yes	Yes	Other	Yes	No	Yes	Yes	Yes	Yes	No	No	Yes	Moderate (8/12)
Brunton, 2018	Cross sectional	Yes	Yes	Other	Yes	No	Yes	Other	Yes	Yes	No	No	Yes	Moderate (7/12)
Hara, 2018	Cross sectional	Yes	Yes	Other	Yes	No	Yes	Yes	Yes	Yes	No	No	Yes	Moderate (8/12)
Takuro, 2018	Cross sectional	Yes	No	Other	Other	No	No	Yes	Yes	Yes	No	No	No	High (4/12)
Sharkey, 1984	Cross sectional	Yes	Yes	Other	Other	No	Yes	Yes	Yes	Yes	No	No	No	Moderate (6/12)
Watanabe, 1991	Cross sectional	Yes	Yes	Other	Other	No	Yes	Yes	No	No	No	No	No	High (4/12)

### 3.6 Main findings

A summary of the findings for each included study is provided in Table 4.

#### 3.6.1 Healthy subjects

Thirteen studies assessed the muscular performance of mouth opening among healthy participants (age ≤65 years; *n* = 1651; 941 females and 710 males). Only three of those studies used a similar measurement device and procedure, and therefore were not appropriate for inclusion in a meta‐analysis. All thirteen studies assessed maximal mouth opening strength, and only one study also measured maximal jaw protrusion strength while none assessed mouth opening endurance. Ten studies that included both males and females found significant sex differences (males more than females), while the other three studies included only males. The maximal mouth opening strength ranged between 7.2 and 36.6 kg for males and 3.4 and 16.4 kg for females. Nine of the thirteen studies were scored as having a moderate risk of bias, three with a high risk of bias and only one^29^ with a low risk of bias.

#### 3.6.2 TMDs

Three studies assessed the muscular performance of mouth opening among patients with TMDs (*n* = 216; 160 females and 74 males). All three used the updated Diagnostic Criteria for Temporomandibular Disorders as the main inclusion criterion.^28^ One study included only patients with pain‐related TMD,^29^ and the other two included patients with pain‐related TMDs and/or intra‐articular TMDs.^30^, ^31^ Each study used different measurement devices and protocols. Two studies measured mouth opening endurance^29^, ^30^ and the other one determined maximal mouth opening strength as an outcome measure.^31^ Two studies compared the muscular performance of patients with pain‐related and/or intra‐articular TMDs to healthy controls,^30^, ^31^ and one study compared the muscular performance of two different pain‐related TMDs subgroups.^29^ Significant reductions of muscular performance were found among patients with TMDs compared to healthy controls, with no difference between TMD subgroups.^30^, ^31^ Patients with TMD‐related pain who presented with “general pain” demonstrated lower endurance compared to those without “general pain”.^29^

### 3.7 Confidence in cumulative evidence

Based upon the GRADE guidelines,^22^ there is only low‐quality evidence to support the findings of mouth opening strength among healthy adults due to the high variability of findings, the different measurement devices and procedures and the lack of reliability and validity. Importantly, there is only very low quality of evidence to support the findings for patients with TMDs due to a very low number of relevant studies, together with the use of different measurement devices and procedures.

## 4 DISCUSSION

This is the first systematic review to comprehensively examine human mouth opener muscle performance. The findings suggest that the parameters of sex and age influence maximal mouth opening strength in healthy population, with large gaps and limitations in the reliability and accuracy of these findings. A very small volume of evidence was found for patients with TMDs. Unlike the availability of information on mouth closer muscles, the evidence regarding the muscular endurance of the mouth opener muscles for both healthy and patient populations is extremely limited.

### 4.1 Healthy adults

As expected, the largest volumes of evidence of mouth opener muscular performance applied to healthy adults who provided the reference data of normal muscular function to which other groups of patients could be compared. However, these data are extremely limited for several reasons, and therefore should be viewed with caution. First, out of the 13 included studies, only one was rated as having a low risk of bias^29^ while three^31^, ^32^, ^33^ were rated as having a high risk and the other nine as having a moderate risk. Second, the variability of maximal mouth opening strength ranged between 7.2 and 36.6 kg for males (average = 29.4 kg) and 3.4 and 16.4 kg for females (average = 13 kg). Such large ranges cannot serve as a reliable and clinically meaningful reference to which the muscular strength of patient groups can be compared. The main reason for this high variability of findings across studies is comparable to the reason which prevented the results of 13 studies to be calculated as a meta‐analysis, specifically, the lack of a similar measurement tool and assessment procedure. Ten different measurement devices were used in those 13 studies, and most of them had different testing protocols. Additionally, only two studies reported the inter‐ and intra‐examiner reliability of their tests as required in such a unique assessment of a relatively under‐researched muscle group. After considering these important methodological weaknesses, a very large strength difference is presented in this review between the sexes. This relatively large strength difference deficit of females versus males is not surprising as it is also present in other muscle groups, such as the grip muscles of the hand and the flexors of the neck.^34^, ^35^

It is also important to note that none of the included 13 studies that assessed healthy adults did not evaluate any aspect of muscular endurance of the mouth openers. These data are especially important for the evaluation of muscular impairment. Other important missing reference data are the performance of the jaw protrusion muscles, which comprise an integral part of the mouth openers,^1^ and none of the included studies assessed either their maximal strength or endurance.

### 4.2 TMDs

Only three studies that assessed the mouth‐opening muscular performance of patients with TMDs were included in this systematic review. This currently available volume of evidence is extremely low for reliable evaluation of the function of this muscle group in TMDs compared to controls. Furthermore, several important limitations were found within this already small volume of relevant evidence. Only one of the three included studies evaluated the maximal mouth opening strength of patients with TMDs^31^ while the other two assessed muscular endurance.^29^, ^30^ Although Ratnayake et al^31^ assessed the reliability of their measurement between repeated attempts during the same session, the required test‐retest for intra‐ and inter‐tester reliability was not performed,^33^ and the results should, therefore, be taken with extra caution. The same methodology for evaluating the reliability of measurement was not performed in the other two studies that evaluated the muscular endurance of patients with TMDs.^29^, ^30^ Finally, none of the studies in this category evaluated the maximal strength of the jaw protrusion muscles which comprise an important component for the physiological mouth opening function.^1^ Based upon the small volume and methodological limitations of evaluating the mouth opening muscular performance of patients with TMDs, the current data cannot serve as a valid reference for functional impairments in this population.

### 4.3 Limitations

The limitations of this study were primarily due to the relatively small volume of available literature. Only fourteen studies met the eligibility criteria of this review, and no homogenic group was found to be appropriate for the meta‐analysis. Only three relevant studies were identified for patients with TMDs.^29^, ^30^, ^31^ This limited volume of relevant clinical evidence precludes the possibility of clinicians who manage patients with TMDs from implementing evidence‐based methodology when considering the mouth‐opening muscular performance as part of their assessment and management. Furthermore, no relevant study for other patient populations, such as those with obstructive sleep apnoea and speech disorders, was found during the literature search.

### 4.4 Future direction

This review highlights the need for future research into several important areas of interest. The most basic scientific need is to establish a valid and reliable measurement device and testing procedure for the maximal strength and endurance capacity of mouth opening muscles (both mandibular depressors and protrusion muscles^1^). This will require a well‐designed intra‐ and inter‐tester reliability study on healthy controls followed by patients with TMDs in order to validate such a test. A proper real‐time observation study on the mouth‐opening muscular performance will be required, probably using a real‐time ultrasonography and/or electromyography devices.

After validating the muscular performance tests, baseline data of healthy controls of different ages will be needed, ideally by performing an international multicentre study. The normal agonist‐antagonist muscular performance ratio between the mouth opener and closer musculature of males and females of different age groups would be another interesting factor for observation at this stage of research, similar to the existing data on different musculoskeletal regions, such as the knee and shoulder.^13^, ^36^, ^37^

The application of the physiological muscular performance data as a reference for comparison with different relevant patient populations in an international multicentre study (TMDs, dysphagia, obstructive sleep apnoea and bruxism) will comprise the next step for investigation. That step may help to identify clinical subgroups that would benefit from muscular rehabilitation programs tailored specifically to improve the mouth‐opening muscular performance.

The clinical implications of the results are to carefully screen for clinical signs and symptoms of the mouth openers in patients with TMDs and to address it during the multidisciplinary rehabilitation process.

## 5 CONCLUSION

This is the first systematic review to comprehensively examine mouth‐opening muscular performance in healthy and TMDs populations. The findings suggest significant influence of the parameters of sex and age, similar to the findings for other muscle groups. This review also exposes several major gaps in the current literature regarding mouth‐opening muscular performance. One is the lack of a valid and reliable test for this unique muscle group, another is the need for an estimation of normal physiological muscular performance and the third is the proper evaluation of muscular performance in patients with common relevant disorders, such as TMD, dysphagia, obstructive sleep apnoea and bruxism.

## CONFLICT OF INTEREST

None.

## AUTHOR'S CONTRIBUTIONS

All authors were involved in study inception and design, and critical manuscript revision. TG collected, analysed and interpreted data and wrote the manuscript. AEP screened papers, extracted data, assessed risk of bias and critically reviewed the manuscript.

### PEER REVIEW

The peer review history for this article is available at https://publons.com/publon/10.1111/joor.13303.

## Data Availability

The data that support the findings of this study are available on request from the corresponding author. The data are not publicly available due to privacy or ethical restrictions.

## APPENDIX 1

Reviewer:

Date:

Study ID:

Title:

Authors:

Lead author contact details:

Country in which the study conducted:

Characteristics of study:

Methods:

Aim of study:

Study design:

Start date:

End date:

Study funding sources:

Possible conflicts of interest for study authors:

Participants:

Population description:

Inclusion criteria:

Exclusion criteria:

Method of recruitment of participants:

Total number of participants:

Diagnostic methods:

Diagnostic tools:

Diagnostic criteria:

Study outcome measures:

Measurement tools:

Testing procedure:

Main findings:

## APPENDIX 2

**Table:**

SIGN	Methodology Checklist 4: Case‐control studies		
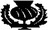
Study identification (Include author, title, year of publication, journal title, pages)			
Guideline topic:		Key Question No:	Reviewer:
Before completing this checklist, consider: 1. Is the paper really a case‐control study? If in doubt, check the study design algorithm available from SIGN and make sure you have the correct checklist. 2. Is the paper relevant to key question? Analyse using PICO (Patient or Population Intervention Comparison Outcome). IF NO REJECT (give reason below). IF YES complete the checklist.			
Reason for rejection: Reason for rejection: 1. Paper not relevant to key question □ 2. Other reason □ (please specify):			
Section 1: Internal validity			
In an well conducted case control study:		Does this study do it?	
1.1	The study addresses an appropriate and clearly focused question.^a^	Yes □ Can't say □	No □
Selection of subjects			
1.2	The cases and controls are taken from comparable populations.^b^	Yes □ Can't say □	No □
1.3	The same exclusion criteria are used for both cases and controls.^c^	Yes □ Can't say □	No □
1.4	What percentage of each group (cases and controls) participated in the study?^d^	Cases: Controls:	
1.5	Comparison is made between participants and non‐participants to establish their similarities or differences.^e^	Yes □ Can't say □	No □
1.6	Cases are clearly defined and differentiated from controls.^f^	Yes □ Can't say □	No □
1.7	It is clearly established that controls are non‐cases.^g^	Yes □ Can't say □	No □
Assessment			
1.8	Measures will have been taken to prevent knowledge of primary exposure influencing case ascertainment.^h^	Yes □ Can't say □	No □ Does not apply □
1.9	Exposure status is measured in a standard, valid and reliable way.^i^	Yes □ Can't say □	No □
Confounding			
1.10	The main potential confounders are identified and taken into account in the design and analysis.^j^	Yes □ Can't say □	No □
Statistical analysis			
1.11	Confidence intervals are provided.^k^	Yes □	No □
Section 2: Overall assessment of the study			
2.1	How well was the study done to minimise the risk of bias or confounding?^l^	High quality (++) □ Acceptable (+) □ Unacceptable – reject 0 □	
2.2	Taking into account clinical considerations, your evaluation of the methodology used, and the statistical power of the study, do you think there is clear evidence of an association between exposure and outcome?	Yes □ Can't say □	No □
2.3	Are the results of this study directly applicable to the patient group targeted by this guideline?	Yes □	No □
2.4	Notes. Summarise the authors conclusions. Add any comments on your own assessment of the study, and the extent to which it answers your question and mention any areas of uncertainty raised above.		

^a^ Unless a clear and well defined question is specified in the report of the review, it will be difficult to assess how well it has met its objectives or how relevant it is to the question you are trying to answer on the basis of the conclusions.

^b^ Study participants may be selected from the target population (all individuals to which the results of the study could be applied), the source population (a defined subset of the target population from which participants are selected), or from a pool of eligible subjects (a clearly defined and counted group selected from the source population. If the study does not include clear definitions of the source population it should be rejected.

^c^ All selection and exclusion criteria should be applied equally to cases and controls. Failure to do so may introduce a significant degree of bias into the results of the study.

^d^ Differences between the eligible population and the participants are important, as they may influence the validity of the study. A participation rate can be calculated by dividing the number of study participants by the number of eligible subjects. It is more useful if calculated separately for cases and controls. If the participation rate is low, or there is a large difference between the two groups, the study results may well be invalid due to differences between participants and non‐participants. In these circumstances, the study should be downgraded, and rejected if the differences are very large.

^e^ Even if participation rates are comparable and acceptable, it is still possible that the participants selected to act as cases or controls may differ from other members of the source population in some significant way. A well conducted case‐control study will look at samples of the non‐participants among the source population to ensure that the participants are a truly representative sample.

^f^ The method of selection of cases is of critical importance to the validity of the study. Investigators have to be certain that cases are truly cases, but must balance this with the need to ensure that the cases admitted into the study are representative of the eligible population. The issues involved in case selection are complex, and should ideally be evaluated by someone with a good understanding of the design of case‐control studies. If the study does not comment on how cases were selected, it is probably safest to reject it as a source of evidence.

^g^ Just as it is important to be sure that cases are true cases, it is important to be sure that controls do not have the outcome under investigation. Control subjects should be chosen so that information on exposure status can be obtained or assessed in a similar way to that used for the selection of cases. If the methods of control selection are not described, the study should be rejected. If different methods of selection are used for cases and controls the study should be evaluated by someone with a good understanding of the design of case‐control studies.

^h^ If there is a possibility that case ascertainment can be influenced by knowledge of exposure status, assessment of any association is likely to be biased. A well conducted study should take this into account in the design of the study.

^i^ The primary outcome measures used should be clearly stated in the study. If the outcome measures are not stated, or the study bases its main conclusions on secondary outcomes, the study should be rejected. Where outcome measures require any degree of subjectivity, some evidence should be provided that the measures used are reliable and have been validated prior to their use in the study.

^j^ Confounding is the distortion of a link between exposure and outcome by another factor that is associated with both exposure and outcome. The possible presence of confounding factors is one of the principal reasons why observational studies are not more highly rated as a source of evidence. The study should indicate which potential confounders have been considered, and how they have been allowed for in the analysis. Clinical judgement should be applied to consider whether all likely confounders have been considered. If the measures used to address confounding are considered inadequate, the study should be downgraded or rejected. A study that does not address the possibility of confounding should be rejected.

^k^ Confidence limits are the preferred method for indicating the precision of statistical results, and can be used to differentiate between an inconclusive study and a study that shows no effect. Studies that report a single value with no assessment of precision should be treated with extreme caution.

^l^ Rate the overall methodological quality of the study, using the following as a guide: High quality (++): Majority of criteria met. Little or no risk of bias. Results unlikely to be changed by further research. Acceptable (+): Most criteria met. Some flaws in the study with an associated risk of bias, Conclusions may change in the light of further studies. Low quality (0): Either most criteria not met, or significant flaws relating to key aspects of study design. Conclusions likely to change in the light of further studies.

## APPENDIX 3

**Table:**

Criteria	Yes	No	Other (CD, NR, NA)
1. Was the research question or objective in this paper clearly stated?			
2. Was the study population clearly specified and defined?			
3. Was the participation rate of eligible persons at least 50%?			
4. Were all the subjects selected or recruited from the same or similar populations (including the same time period)? Were inclusion and exclusion criteria for being in the study prespecified and applied uniformly to all participants?			
5. Was a sample size justification, power description, or variance and effect estimates provided?			
6. For the analyses in this paper, were the exposure(s) of interest measured prior to the outcome(s) being measured?			
7. Was the timeframe sufficient so that one could reasonably expect to see an association between exposure and outcome if it existed?			
8. For exposures that can vary in amount or level, did the study examine different levels of the exposure as related to the outcome (e.g., categories of exposure, or exposure measured as continuous variable)?			
9. Were the exposure measures (independent variables) clearly defined, valid, reliable, and implemented consistently across all study participants?			
10. Was the exposure(s) assessed more than once over time?			
11. Were the outcome measures (dependent variables) clearly defined, valid, reliable, and implemented consistently across all study participants?			
12. Were the outcome assessors blinded to the exposure status of participants?			
13. Was loss to follow‐up after baseline 20% or less?			
14. Were key potential confounding variables measured and adjusted statistically for their impact on the relationship between exposure(s) and outcome(s)?			

**Table:**

Quality Rating (Good, Fair, or Poor)
Rater #1 initials:
Rater #2 initials:
Additional Comments (If POOR, please state why):

CD, cannot determine; NA, not applicable; NR, not reported.

### GUIDANCE FOR ASSESSING THE QUALITY OF OBSERVATIONAL COHORT AND CROSS‐SECTIONAL STUDIES

The guidance document below is organized by question number from the tool for quality assessment of observational cohort and cross‐sectional studies.

### QUESTION 1. RESEARCH QUESTION

Did the authors describe their goal in conducting this research? Is it easy to understand what they were looking to find? This issue is important for any scientific paper of any type. Higher quality scientific research explicitly defines a research question.

### QUESTIONS 2 AND 3. STUDY POPULATION

Did the authors describe the group of people from which the study participants were selected or recruited, using demographics, location, and time period? If you were to conduct this study again, would you know who to recruit, from where, and from what time period? Is the cohort population free of the outcomes of interest at the time they were recruited?

An example would be men over 40 years old with type 2 diabetes who began seeking medical care at Phoenix Good Samaritan Hospital between January 1, 1990 and December 31, 1994. In this example, the population is clearly described as: (1) who (men over 40 years old with type 2 diabetes); (2) where (Phoenix Good Samaritan Hospital); and (3) when (between January 1, 1990 and December 31, 1994). Another example is women ages 34 to 59 years of age in 1980 who were in the nursing profession and had no known coronary disease, stroke, cancer, hypercholesterolemia, or diabetes, and were recruited from the 11 most populous States, with contact information obtained from State nursing boards.

In cohort studies, it is crucial that the population at baseline is free of the outcome of interest. For example, the nurses’ population above would be an appropriate group in which to study incident coronary disease. This information is usually found either in descriptions of population recruitment, definitions of variables, or inclusion/exclusion criteria.

You may need to look at prior papers on methods in order to make the assessment for this question. Those papers are usually in the reference list.

If fewer than 50% of eligible persons participated in the study, then there is concern that the study population does not adequately represent the target population. This increases the risk of bias.

### QUESTION 4. GROUPS RECRUITED FROM THE SAME POPULATION AND UNIFORM ELIGIBILITY CRITERIA

Were the inclusion and exclusion criteria developed prior to recruitment or selection of the study population? Were the same underlying criteria used for all of the subjects involved? This issue is related to the description of the study population, above, and you may find the information for both of these questions in the same section of the paper.

Most cohort studies begin with the selection of the cohort; participants in this cohort are then measured or evaluated to determine their exposure status. However, some cohort studies may recruit or select exposed participants in a different time or place than unexposed participants, especially retrospective cohort studies–which is when data are obtained from the past (retrospectively), but the analysis examines exposures prior to outcomes. For example, one research question could be whether diabetic men with clinical depression are at higher risk for cardiovascular disease than those without clinical depression. So, diabetic men with depression might be selected from a mental health clinic, while diabetic men without depression might be selected from an internal medicine or endocrinology clinic. This study recruits groups from different clinic populations, so this example would get a "no."

However, the women nurses described in the question above were selected based on the same inclusion/exclusion criteria, so that example would get a "yes."

### QUESTION 5. SAMPLE SIZE JUSTIFICATION

Did the authors present their reasons for selecting or recruiting the number of people included or analyzed? Do they note or discuss the statistical power of the study? This question is about whether or not the study had enough participants to detect an association if one truly existed.

A paragraph in the methods section of the article may explain the sample size needed to detect a hypothesized difference in outcomes. You may also find a discussion of power in the discussion section (such as the study had 85 percent power to detect a 20 percent increase in the rate of an outcome of interest, with a 2‐sided alpha of 0.05). Sometimes estimates of variance and/or estimates of effect size are given, instead of sample size calculations. In any of these cases, the answer would be "yes."

However, observational cohort studies often do not report anything about power or sample sizes because the analyses are exploratory in nature. In this case, the answer would be "no." This is not a "fatal flaw." It just may indicate that attention was not paid to whether the study was sufficiently sized to answer a prespecified question–i.e., it may have been an exploratory, hypothesis‐generating study.

### QUESTION 6. EXPOSURE ASSESSED PRIOR TO OUTCOME MEASUREMENT

This question is important because, in order to determine whether an exposure causes an outcome, the exposure must come before the outcome.

For some prospective cohort studies, the investigator enrolls the cohort and then determines the exposure status of various members of the cohort (large epidemiological studies like Framingham used this approach). However, for other cohort studies, the cohort is selected based on its exposure status, as in the example above of depressed diabetic men (the exposure being depression). Other examples include a cohort identified by its exposure to fluoridated drinking water and then compared to a cohort living in an area without fluoridated water, or a cohort of military personnel exposed to combat in the Gulf War compared to a cohort of military personnel not deployed in a combat zone.

With either of these types of cohort studies, the cohort is followed forward in time (i.e., prospectively) to assess the outcomes that occurred in the exposed members compared to nonexposed members of the cohort. Therefore, you begin the study in the present by looking at groups that were exposed (or not) to some biological or behavioral factor, intervention, etc., and then you follow them forward in time to examine outcomes. If a cohort study is conducted properly, the answer to this question should be "yes," since the exposure status of members of the cohort was determined at the beginning of the study before the outcomes occurred.

For retrospective cohort studies, the same principal applies. The difference is that, rather than identifying a cohort in the present and following them forward in time, the investigators go back in time (i.e., retrospectively) and select a cohort based on their exposure status in the past and then follow them forward to assess the outcomes that occurred in the exposed and nonexposed cohort members. Because in retrospective cohort studies the exposure and outcomes may have already occurred (it depends on how long they follow the cohort), it is important to make sure that the exposure preceded the outcome.

Sometimes cross‐sectional studies are conducted (or cross‐sectional analyses of cohort‐study data), where the exposures and outcomes are measured during the same timeframe. As a result, cross‐sectional analyses provide weaker evidence than regular cohort studies regarding a potential causal relationship between exposures and outcomes. For cross‐sectional analyses, the answer to Question 6 should be "no."

### QUESTION 7. SUFFICIENT TIMEFRAME TO SEE AN EFFECT

Did the study allow enough time for a sufficient number of outcomes to occur or be observed, or enough time for an exposure to have a biological effect on an outcome? In the examples given above, if clinical depression has a biological effect on increasing risk for CVD, such an effect may take years. In the other example, if higher dietary sodium increases BP, a short timeframe may be sufficient to assess its association with BP, but a longer timeframe would be needed to examine its association with heart attacks.

The issue of timeframe is important to enable meaningful analysis of the relationships between exposures and outcomes to be conducted. This often requires at least several years, especially when looking at health outcomes, but it depends on the research question and outcomes being examined.

Cross‐sectional analyses allow no time to see an effect, since the exposures and outcomes are assessed at the same time, so those would get a "no" response.

### QUESTION 8. DIFFERENT LEVELS OF THE EXPOSURE OF INTEREST

If the exposure can be defined as a range (examples: drug dosage, amount of physical activity, amount of sodium consumed), were multiple categories of that exposure assessed? (for example, for drugs: not on the medication, on a low dose, medium dose, high dose; for dietary sodium, higher than average U.S. consumption, lower than recommended consumption, between the two). Sometimes discrete categories of exposure are not used, but instead exposures are measured as continuous variables (for example, mg/day of dietary sodium or BP values).

In any case, studying different levels of exposure (where possible) enables investigators to assess trends or dose‐response relationships between exposures and outcomes–e.g., the higher the exposure, the greater the rate of the health outcome. The presence of trends or dose‐response relationships lends credibility to the hypothesis of causality between exposure and outcome.

For some exposures, however, this question may not be applicable (e.g., the exposure may be a dichotomous variable like living in a rural setting versus an urban setting, or vaccinated/not vaccinated with a one‐time vaccine). If there are only two possible exposures (yes/no), then this question should be given an "NA," and it should not count negatively towards the quality rating.

### QUESTION 9. EXPOSURE MEASURES AND ASSESSMENT

Were the exposure measures defined in detail? Were the tools or methods used to measure exposure accurate and reliable–for example, have they been validated or are they objective? This issue is important as it influences confidence in the reported exposures. When exposures are measured with less accuracy or validity, it is harder to see an association between exposure and outcome even if one exists. Also as important is whether the exposures were assessed in the same manner within groups and between groups; if not, bias may result.

For example, retrospective self‐report of dietary salt intake is not as valid and reliable as prospectively using a standardized dietary log plus testing participants’ urine for sodium content. Another example is measurement of BP, where there may be quite a difference between usual care, where clinicians measure BP however it is done in their practice setting (which can vary considerably), and use of trained BP assessors using standardized equipment (e.g., the same BP device which has been tested and calibrated) and a standardized protocol (e.g., patient is seated for 5 minutes with feet flat on the floor, BP is taken twice in each arm, and all four measurements are averaged). In each of these cases, the former would get a "no" and the latter a "yes."

Here is a final example that illustrates the point about why it is important to assess exposures consistently across all groups: If people with higher BP (exposed cohort) are seen by their providers more frequently than those without elevated BP (nonexposed group), it also increases the chances of detecting and documenting changes in health outcomes, including CVD‐related events. Therefore, it may lead to the conclusion that higher BP leads to more CVD events. This may be true, but it could also be due to the fact that the subjects with higher BP were seen more often; thus, more CVD‐related events were detected and documented simply because they had more encounters with the health care system. Thus, it could bias the results and lead to an erroneous conclusion.

### QUESTION 10. REPEATED EXPOSURE ASSESSMENT

Was the exposure for each person measured more than once during the course of the study period? Multiple measurements with the same result increase our confidence that the exposure status was correctly classified. Also, multiple measurements enable investigators to look at changes in exposure over time, for example, people who ate high dietary sodium throughout the followup period, compared to those who started out high then reduced their intake, compared to those who ate low sodium throughout. Once again, this may not be applicable in all cases. In many older studies, exposure was measured only at baseline. However, multiple exposure measurements do result in a stronger study design.

### QUESTION 11. OUTCOME MEASURES

Were the outcomes defined in detail? Were the tools or methods for measuring outcomes accurate and reliable–for example, have they been validated or are they objective? This issue is important because it influences confidence in the validity of study results. Also important is whether the outcomes were assessed in the same manner within groups and between groups.

An example of an outcome measure that is objective, accurate, and reliable is death–the outcome measured with more accuracy than any other. But even with a measure as objective as death, there can be differences in the accuracy and reliability of how death was assessed by the investigators. Did they base it on an autopsy report, death certificate, death registry, or report from a family member? Another example is a study of whether dietary fat intake is related to blood cholesterol level (cholesterol level being the outcome), and the cholesterol level is measured from fasting blood samples that are all sent to the same laboratory. These examples would get a "yes." An example of a "no" would be self‐report by subjects that they had a heart attack, or self‐report of how much they weigh (if body weight is the outcome of interest).

Similar to the example in Question 9, results may be biased if one group (e.g., people with high BP) is seen more frequently than another group (people with normal BP) because more frequent encounters with the health care system increases the chances of outcomes being detected and documented.

### QUESTION 12. BLINDING OF OUTCOME ASSESSORS

Blinding means that outcome assessors did not know whether the participant was exposed or unexposed. It is also sometimes called "masking." The objective is to look for evidence in the article that the person(s) assessing the outcome(s) for the study (for example, examining medical records to determine the outcomes that occurred in the exposed and comparison groups) is masked to the exposure status of the participant. Sometimes the person measuring the exposure is the same person conducting the outcome assessment. In this case, the outcome assessor would most likely not be blinded to exposure status because they also took measurements of exposures. If so, make a note of that in the comments section.

As you assess this criterion, think about whether it is likely that the person(s) doing the outcome assessment would know (or be able to figure out) the exposure status of the study participants. If the answer is no, then blinding is adequate. An example of adequate blinding of the outcome assessors is to create a separate committee, whose members were not involved in the care of the patient and had no information about the study participants’ exposure status. The committee would then be provided with copies of participants’ medical records, which had been stripped of any potential exposure information or personally identifiable information. The committee would then review the records for prespecified outcomes according to the study protocol. If blinding was not possible, which is sometimes the case, mark "NA" and explain the potential for bias.

### QUESTION 13. FOLLOWUP RATE

Higher overall followup rates are always better than lower followup rates, even though higher rates are expected in shorter studies, whereas lower overall followup rates are often seen in studies of longer duration. Usually, an acceptable overall followup rate is considered 80 percent or more of participants whose exposures were measured at baseline. However, this is just a general guideline. For example, a 6‐month cohort study examining the relationship between dietary sodium intake and BP level may have over 90 percent followup, but a 20‐year cohort study examining effects of sodium intake on stroke may have only a 65 percent followup rate.

### QUESTION 14. STATISTICAL ANALYSES

Were key potential confounding variables measured and adjusted for, such as by statistical adjustment for baseline differences? Logistic regression or other regression methods are often used to account for the influence of variables not of interest.

This is a key issue in cohort studies, because statistical analyses need to control for potential confounders, in contrast to an RCT, where the randomization process controls for potential confounders. All key factors that may be associated both with the exposure of interest and the outcome–that are not of interest to the research question–should be controlled for in the analyses.

For example, in a study of the relationship between cardiorespiratory fitness and CVD events (heart attacks and strokes), the study should control for age, BP, blood cholesterol, and body weight, because all of these factors are associated both with low fitness and with CVD events. Well‐done cohort studies control for multiple potential confounders.

### SOME GENERAL GUIDANCE FOR DETERMINING THE OVERALL QUALITY RATING OF OBSERVATIONAL COHORT AND CROSS‐SECTIONAL STUDIES

The questions on the form are designed to help you focus on the key concepts for evaluating the internal validity of a study. They are not intended to create a list that you simply tally up to arrive at a summary judgment of quality.

Internal validity for cohort studies is the extent to which the results reported in the study can truly be attributed to the exposure being evaluated and not to flaws in the design or conduct of the study–in other words, the ability of the study to draw associative conclusions about the effects of the exposures being studied on outcomes. Any such flaws can increase the risk of bias.

Critical appraisal involves considering the risk of potential for selection bias, information bias, measurement bias, or confounding (the mixture of exposures that one cannot tease out from each other). Examples of confounding include co‐interventions, differences at baseline in patient characteristics, and other issues throughout the questions above. High risk of bias translates to a rating of poor quality. Low risk of bias translates to a rating of good quality. (Thus, the greater the risk of bias, the lower the quality rating of the study.)

In addition, the more attention in the study design to issues that can help determine whether there is a causal relationship between the exposure and outcome, the higher quality the study. These include exposures occurring prior to outcomes, evaluation of a dose‐response gradient, accuracy of measurement of both exposure and outcome, sufficient timeframe to see an effect, and appropriate control for confounding–all concepts reflected in the tool.

Generally, when you evaluate a study, you will not see a "fatal flaw," but you will find some risk of bias. By focusing on the concepts underlying the questions in the quality assessment tool, you should ask yourself about the potential for bias in the study you are critically appraising. For any box where you check "no" you should ask, "What is the potential risk of bias resulting from this flaw in study design or execution?" That is, does this factor cause you to doubt the results that are reported in the study or doubt the ability of the study to accurately assess an association between exposure and outcome?

The best approach is to think about the questions in the tool and how each one tells you something about the potential for bias in a study. The more you familiarize yourself with the key concepts, the more comfortable you will be with critical appraisal. Examples of studies rated good, fair, and poor are useful, but each study must be assessed on its own based on the details that are reported and consideration of the concepts for minimizing bias.
